# Measurement properties of smartphone approaches to assess key lifestyle behaviours: protocol of a systematic review

**DOI:** 10.1186/s13643-020-01375-w

**Published:** 2020-06-03

**Authors:** Louise Thornton, Bridie Osman, Annie B. Wescott, Matthew Sunderland, Katrina Champion, Olivia Green, Frances Kay-Lambkin, Tim Slade, Nickie Newton, Cath Chapman, Maree Teesson, Katherine Mills, Louise Birrell, David Lubans, Pepijn Van de Ven, John Torous, Belinda Parmenter, Lauren Gardner, Health4Life team

**Affiliations:** 1 The Matilda Centre, The University of Sydney, Sydney, New South Wales Australia; 2 Galter Health Sciences Library & Learning Center, Northwestern University Feinberg School of Medicine, Chicago, IL USA; 3 Priority Research Centre for Brain and Mental Health, The University of Newcastle, Newcastle, New South Wales Australia; 4 Priority Research Centre for Physical Activity and Nutrition, The University of Newcastle, Newcastle, New South Wales Australia; 5 Department of Electronics and Computer Engineering, The University of Limerick, Limerick, Ireland; 6 Beth Israel Deaconness Medical Center, Harvard Medical School, Boston, MA USA; 7 School of Medical Sciences, The University of New South Wales, Sydney, New South Wales Australia

**Keywords:** Smartphone, App, Health, mHealth, Prevention, Risk, Alcohol, Smoking, Diet, Physical activity, Sedentary behaviour, Sleep

## Abstract

**Background:**

Six core behavioural risk factors (poor diet, physical activity, sedentary behaviour, alcohol misuse, smoking and unhealthy sleep patterns) have been identified as strong determinants of chronic disease, such as cardiovascular disease, diabetes and cancers. Smartphones have the potential to provide a real-time, pervasive, unobtrusive and cost-effective way to measure health behaviours and deliver instant feedback to users. Despite this, validity of using smartphones to measure these six key behaviours is largely unknown. The proposed systematic review aims to address this gap by identifying existing smartphone-based approaches to measure these health behaviours and critically appraising, comparing and summarizing the quality of their measurement properties.

**Methods:**

A systematic search of the Ovid MEDLINE, Embase (Elsevier), Cochrane Library (Wiley), PsychINFO (EBSCOhost), CINAHL (EBSCOHost), Web of Science (Clarivate), SPORTDiscus (EBSCOhost) and IEEE Xplore Digital Library databases will be conducted from January 2007 to March 2020. Eligible studies will be those written in English that measure at least one of the six health behaviours of interest via a smartphone and report on at least one measurement property. The primary outcomes will be validity, reliability and/or responsiveness of these measurement approaches. A secondary outcome will be the feasibility (e.g. user burden, usability and cost) of identified approaches. No restrictions will be placed on the participant population or study design. Two reviewers will independently screen studies for eligibility, extract data and assess the risk of bias. The study methodological quality (or bias) will be appraised using an appropriate tool. Our results will be described in a narrative synthesis. If feasible, random effects meta-analysis will be conducted where appropriate.

**Discussion:**

The results from this review will provide important information about the types of smartphone-based approaches currently available to measure the core behavioural risk factors for chronic disease and the quality of their measurement properties. It will allow recommendations on the most suitable and effective measures of these lifestyle behaviours using smartphones. Valid and reliable measurement of these behaviours and risk factor opens the door to targeted and real-time delivery of health behaviour interventions, providing unprecedented opportunities to offset the trajectory toward chronic disease.

**Systematic review registration:**

PROSPERO: CRD42019122242

## Background

Chronic diseases are among the most costly and harmful worldwide and are currently the leading cause of death and disability. Cancers, cardiovascular diseases (CVD) and diabetes are among some of the most prevalent of these chronic diseases [1]. In 2017, CVD alone accounted for nearly 30% of all deaths in Australia—deaths that were largely preventable [2]. In the same year, the European Heart Network reported that in Europe, CVD accounted for 47% of all deaths [3]. Poor diet, physical inactivity, alcohol use and smoking have long been recognised as key behavioural risks associated with chronic disease and life expectancy lost [4–6]. In more recent years, sedentary behaviour (sitting and screen time) [7, 8] and unhealthy sleep patterns [9] have emerged as significant contributors to the onset of chronic disease (i.e. ‘the Big 6’). Valid measurement and self-monitoring of these risk behaviours are central to successful chronic disease risk-reduction interventions, and meta-analyses provide evidence for the efficacy of self-monitoring of diet, physical activity, weight, and tobacco and alcohol use towards this end [10–13]. However, measurement of many of these health behaviours can be difficult and burdensome to participants. Traditional measurement techniques using pen-and-paper and in-person assessment of health behaviours are also often subject to problems such as recall bias, with respondents forgetting to record data or recording information incorrectly or losing information [14]. Alternate measurement techniques are needed to try to increase compliance and accuracy with recording data, reduce respondent burden and increase the quality and detail of health behaviour information it is possible to collect.

Smartphones have become an integral part of many people’s lives, with 45.12% of the global population in 2020 [15] and 81% of the US adult population in 2019 [16] owning a smartphone. Smartphone applications (apps) and wearable devices are often used daily by individuals to record and measure a wide range of health behaviours [17]. However, currently, smartphones are the most common way to self-monitor health behaviours, as ownership rates of wearable devices remain comparatively low, with only 10.5% of the global population and 21% of the US adult population [18] owning a wearable device in 2019 [19]. As of 2017, there were over 318,000 smartphone health apps available on the major app stores, many of which allow users to record or measure their health behaviours, with this number continuing to grow by over 200 apps every day [20].

Innovations in technology have more recently seen a wide range of sensors (accelerometers, gyroscopes, light sensors, GPS and magnetometers) incorporated into smartphones as standard features, which makes the smartphone capable of continuously monitoring users’ context (e.g. physical activity, location and environment). The data generated by these sensors and participants’ phone use (e.g. if the phone screen is on or off, in-phone communication and gestures used) can be collected ‘passively’, without the active involvement of the phone user, and can be used to generate information about phone users’ behaviours [21]. This allows opportunities for more in-depth, accurate and less invasive data collection of health behaviours. These approaches also provide opportunities to objectively measure some behaviours that previously have been too difficult or burdensome for participants to measure (e.g. physical activity and sleep) and could help to identify behaviours that are being engaged in at levels considered risky to short- and long-term health and wellbeing.

Smartphone technologies also provide opportunities for ‘just-in-time’ adaptive interventions where tailored support can be provided ‘in the moment’ to participants, allowing the relevant support material to be delivered to a person at the time and in a context when it is most salient [22]. For example, the same app that passively monitors user physical activity over time could also be programmed to provide a brief, motivational intervention to increase activity, if that physical activity drops below a pre-defined level for that user. With increasing evidence suggesting both the preventative and risk-reduction effects of exercise, sleep and diet on mental health [23–27], the potential for such ‘closed loop systems’ that both monitor and intervene is high and broader than the target behaviour or activity that is being monitored. However, accurately measuring key lifestyle behaviours in real time using smartphones is hampered by a lack of understanding of the validity of smartphones to measure these behaviours, inducing a lack of consensus around which behaviours are most important to monitor (what), which sensors are the most reliable to monitor (how) and what behaviour change thresholds warrant an intervention (when). Moreover, there is no consensus on how raw sensor data should be translated to a higher level metric. The lifestyle behaviours of interest in the proposed review are highly complex, and it is unlikely that any one individual sensor will be able to act as a reliable and accurate proxy for the behaviour of interest.

To address current knowledge gaps, the proposed review aims to systematically identify and evaluate the existing literature reporting on the measurement properties of smartphone-based approaches to measure diet, physical activity, sedentary behaviour (sitting and screen time), alcohol use, tobacco use and sleep. The specific objectives of this review are to:

1. Identify and describe the ways in which tobacco use, alcohol use, physical activity, diet, sedentary behaviour and sleep patterns have been measured using smartphone technology among populations of any age, gender or health status
2. Describe and critically evaluate the available evidence on the measurement properties, with attention also paid to the feasibility and usability of these measurement approaches
3. Provide recommendations on the most suitable and effective ways of measuring tobacco use, alcohol misuse, physical activity, diet, sedentary behaviour and sleep patterns using smartphones

## Methods

This systematic review has been registered with the International Prospective Register of Systematic Reviews (PROSPERO: CRD42019122242). This systematic review protocol was written in accordance with the Preferred Reporting Items for Systematic Review and Meta-Analysis Protocols (PRISMA-P) guidelines (see additional file 1) [28]. The systematic review itself will also be written in accordance with the PRISMA statement.

### Eligibility criteria

Published studies with any type of study design, involving participants of any age, gender, geographical area and health status, were eligible for inclusion in the proposed review. To be eligible for inclusion, published studies needed to be written in English, published after 2007, and describe a smartphone-based approach to measuring at least one of the following behaviours via a smartphone: tobacco use, alcohol use, physical activity, diet, sedentary (sitting time and screen time) and sleep patterns. Studies were required to report on at least one measurement property identified in the COSMIN Taxonomy of Measurement Properties (see Table 1).

**Table 1:** COSMIN taxonomy of measurement properties [29]

**Domain**	**Measurement properties**	**Definition**
**Reliability** (The degree to which the measurement is free from measurement error)	Internal consistency	The degree of the interrelatedness among the items
	Reliability	The proportion of the total variance in the measurements which is due to ‘true’ differences between patients
	Measurement error	The systematic and random error of a patient’s score that is not attributed to true changes in the construct to be measured
**Validity** (The degree to which an instrument measures the construct(s) it purports to measure)	Content validity (including face validity)	The degree to which the content of an instrument is an adequate reflection of the construct to be measured
	Construct validity (including structural validity, hypothesis testing, cross-cultural validity)	The degree to which the scores of an instrument are consistent with hypotheses (for instance, with regard to internal relationships, relationships to scores of other instruments or differences between relevant groups) based on the assumption that the instrument validly measures the construct to be measured
	Criterion validity	The degree to which the scores of an instrument are an adequate reflection of a ‘gold standard’
**Responsiveness**	Responsiveness	The ability of an instrument to detect change over time in the construct to be measured

Studies were excluded from the systematic review if they describe only the feasibility of the measurement approach without describing at least one other measurement property. Similarly, studies that describe the methodological effectiveness of a wearable device (e.g. Fitbit) alone or a wearable device integrated with a smartphone in a way that does not complement its functionality (e.g. studies describing cases where the smartphone is used only to gather data from the device and send it to a server) were not included. Studies that measure physical activity as mobility or technique only were not included. Finally, studies that describe the methodological effectiveness of using text messaging or a website only to measure health behaviours were also not included.

### Search strategy

A research librarian developed a database search strategy in consultation with members of the review team. The following electronic databases will be searched (from January 2007 to March 2020): Ovid MEDLINE, Embase (Elsevier), Cochrane Library (Wiley), PsychINFO (EBSCOhost), CINAHL (EBSCOHost), Web of Science (Clarivate), SPORTDiscus (EBSCOhost) and IEEE Xplore Digital Library. The search will be limited to English language and studies involving human subjects. Only studies published after 2007 will be included in the review. The start date of 2007 was chosen as it is the year in which the first mobile phones with large capacitive touchscreens using direct finger input, as opposed to a stylus or keypad, were released. The search strategy will involve the use of commonly used database-specific subject headings and free-text keywords appearing in the title and abstract across three search groups. The first search group will contain terms for ‘the Big 6’ (i.e. tobacco use, alcohol use, physical activity, diet, sedentary behaviour/recreational screen time and sleep patterns). The second group will contain terms associated with smartphones, while the third group contained search terms related to methodological effectiveness. The three search groups will be combined using Boolean operators to identify studies and review articles examining the methodological effectiveness of using smartphones to measure at least one of ‘the Big 6’ health behaviours. The search terms will be initially developed for MEDLINE and adapted for the other databases. A draft search strategy for MEDLINE is provided as additional file (see additional file 2). The searches will be supplemented by cross-checking reference lists of relevant and key publications. All papers identified in the search strategy will be exported into a citation management system (Endnote) for de-duplication and uploaded to the Covidence online software program for screening. Prior to extracting data from the references, the reference lists of eligible papers will be reviewed to identify other relevant studies, and recent related systematic reviews will be consulted to identify any additional studies.

### Data extraction and screening

The Covidence online program will be used to manage records and data throughout the review. Articles will be screened by one reviewer using Covidence against the inclusion and exclusion criteria using titles and abstracts. Those articles that meet the criteria based on title and abstract will have the full text double screened, according to the eligibility criteria, by two of seven members of the research team blinded to each other’s ratings. Any full-text articles unavailable to the research team through the university resources will be requested through inter-library loans or the corresponding author. Inter-reviewer agreement will be reported. Any disagreements will be resolved with the assistance of a third researcher.

Preliminary data will be screened, as per the protocol, and extracted by two reviewers blinded to each other’s ratings, from a random sample of 10 full-text articles based upon an initial data extraction plan. Based on this pilot, the data extraction plan was adjusted and the following plan will be applied to all included full-text articles:

1) *Publication details (author, year of publication, citation and location)*

*2) Study characteristics (sample size, age [mean, SD, range], gender balance, study setting, study design and study objective)*

*3) Health behaviour/s measured*

*4) Approach used to measure the health behaviour:*

- *If the measure is based on self-report or an objective measure of the health behaviour*
- *If the data is collected actively or passively*
- *Type of phone used and operating system*
- *Details of the mobile application used to measure the health behaviour*
- *Any specific phone sensors used to collect information about the behaviour*
- *If a wearable device was used in conjunction with the smartphone*
- *Available details regarding the algorithm used to compute the behaviour, sampling frequency, filtering applied and phone location during measurement*
- *If the mobile app, sensors, algorithms, etch is open source*

*5) Approach used to assess the measurement properties of the approach (including all measurement properties identified in the COSMIN Taxonomy of Measurement Properties)*

*6) Findings regarding the measurement properties of the approach*

*7) Findings regarding the feasibility of the approach (e.g. compliance, usability, participant burden, participant feedback, cost to participant and incentive/reimbursement)*

*8) Any other findings or implications:*

- *Details of any comparison measures*
- *Abbreviations used*
- *Any conflicts of interest*

Where necessary, the corresponding author of included studies will be contacted by email to obtain any required data not presented in the published paper.

### Outcomes

The primary outcomes of this review will be the measurement properties of smartphone-based approaches to assess the six key lifestyle behaviours of interest. The specific measurement properties to be investigated (as reported) have been drawn from the COSMIN Taxonomy of Measurement Properties [29]. They include internal consistency, reliability, measurement error, content validity (including face validity), construct validity (including structural validity and cross-cultural validity), criterion validity and responsiveness. The feasibility (including, but not limited to compliance, user burden, usability and cost) of the identified measurement approaches will also be assessed as a secondary outcome.

### Risk of bias

Data assessing the quality and risk of bias for each study will also be extracted. Two reviewers will independently assess the risk of bias of the included studies using the COSMIN Risk of Bias checklist [30]. Each measurement property reported, within the relevant section of the COSMIN risk of bias checklist, will be completed, and studies will receive a quality rating for that particular measurement property. Agreement between reviewers will be assessed using Cohen’s kappa statistic. Any conflict between the two raters will be resolved by a third reviewer. If sufficient data is available, publication bias will be examined by inspection of funnel plots for asymmetry, and the quality of the body of evidence will be assessed using the Grading of Recommendations Assessment, Development and Evaluation (GRADE) framework.

### Analysis

The findings regarding the measurement properties of the approaches identified will be grouped according to the health behaviour measured. Where enough consistent data exists for a particular approach to measure a health behaviour, a meta-analysis using the random effects model [31] will be undertaken to provide a quantitative synthesis of the study findings and I^2^ statistic [32] and Cochran’s Q test [33] to quantify any heterogeneity. It is anticipated however, that the data collected will be too diverse to meet the threshold for a meta-analytic approach, and in that case, a narrative synthesis method will be undertaken. Where appropriate, outcomes will be described separately for self-report and objective measurement approaches, adolescents and young adults (i.e. 10–24 years) and adults (25 years +) as well as different study designs. If sufficient data is available, subgroup analyses will be conducted to examine these variables as potential sources of heterogeneity. If additional covariates are identified and included in additional analyses, these will be reported in the final review.

One aspect of targeted improvements is an analysis of the combination of sensors used to measure the health behaviours of interest. Such an analysis may allow us to provide recommendations as to what sensors should be used for the measurement of any of the big six health behaviours. We endeavour to analyse how best to combine these sensors to allow their respective outputs to inform on the behaviour of interest to the highest degree possible. Where this information is provided, we will also take into consideration technical details such as sampling frequency and data pre-processing (such as filtering) applied.

## Discussion

The proposed systematic review will be the first to bring together the existing evidence of the measurement properties of smartphone-based approaches to measure key lifestyle behaviours associated with increased chronic disease risk. This systematic review will inform the future use of smartphone apps to measure key lifestyle behaviours by identifying which existing approaches are more methodologically valid and reliable as well as collecting information about the nature of these approaches, including if the approach is based on self-report or objective measurement, type of device used and the specific sensors and algorithms used to calculate behaviours. This detailed information will help to inform how these measurement approaches could be implemented by clinicians to help clients better measure and manage their health behaviours, improve measurement of these behaviours in research settings and inform the development of interventions requiring accurate real-time measurement of lifestyle behaviours, such as just-in-time adaptive interventions, which have the potential to provide unprecedented opportunities to offset the trajectory toward chronic disease. Of particular interest are any methodologically sound approaches that minimise participant burden and increase compliance in outcome collection during these measurements.

This review will also attempt to investigate the use of the combination of smartphone sensors to measure health behaviours. Our results may allow us to provide recommendations as to what sensors should be used for the measurement of any of the six health behaviours. Where appropriate information is provided, we will also examine and provide recommendations regarding technical details such as sampling frequency and data pre-processing (such as filtering).

Realizing that most digital health-related studies have not been replicated, we will seek evidence for methods and tools that offer replicability. We hope to identify replicable studies but are aware that these may not exist. Thus, we will also identify apps and sensors that are open source, offer access to underlying analysis code, and are able to easily be implemented in new settings. This review will help identify those tools that will be of the greatest interest to the field today.

To date, little is understood about how the use of these digital health tools varies by age and country. As social norms around technology use, privacy considerations and access to digital devices may vary by population and age, our review will offer information on global trends in this space. Understanding which age groups are most likely to engage is an important and open question in the field, with some concern that younger people may be already so active on their smartphones that they may not pay as much attention to any new app or program. On the other hand, such digitally skilled youth may also be the ideal population to use these new tools as they already possess the knowledge and skills to optimally use them.

Despite these strengths, it is important to acknowledge some inherent limitations of our proposed approach. Firstly, by limiting our search to English language papers, we are potentially missing other smartphone-based measurement approaches discussed in the non-English literature. Unfortunately, it is beyond the expertise and resources of this review team to include articles not written in English. Secondly, due to time and resource constraints, a decision to only double-screen articles at the full-text review stage was made, meaning there is a risk that some potentially relevant articles may have been screened out at the earlier stages of the review. However, in an attempt to mitigate this limitation, all reviewers participated in training sessions where multiple reviewers independently reviewed and discussed the same selection of articles to help ensure consistency across reviewers. Finally, information regarding how apps measure the behaviours of interest, and their accuracy, is commercial-in-confidence for many commercially developed apps. For this reason, this review will not be able to comment on the measurement properties of approaches used by the large number of publicly available apps which purport to measure the behaviours of interest, but for which no evaluations have been reported in the peer-reviewed literature.

The review will aim to provide recommendations regarding which currently available mobile-based self-report measures and smartphone-based objective measurement approaches are the most suitable and effective ways to measure each of the six behaviours of interest based on the current evidence of their measurement properties. It will also identify any gaps in the existing literature and areas for improvement which could help to inform the development of new smartphone-based measurement tools.

## Supplementary information

**Additional file 1.** PRISMA-P checklist.

**Additional file 2.** MEDLINE search.

## Data Availability

Not applicable
