# Supplementary material for: Measurement properties of smartphone approaches to assess key lifestyle behaviours: protocol of a systematic review
Source: Syst Rev. 2020 Jun 3;9:127. doi: 10.1186/s13643-020-01375-w (PMC7271443; doi:10.1186/s13643-020-01375-w)
Supplement: Supplementary file 2 — Additional file 2. MEDLINE search. [file 13643_2020_1375_MOESM2_ESM.docx]

**Additional File 2. Medline Search Strategy**

| **#** | **Searches** |
| --- | --- |
| 1 | exp Exercise/ |
| 2 | exp sports/ |
| 3 | exp dancing/ |
| 4 | exp physical exertion/ |
| 5 | recreation/ |
| 6 | exp healthy lifestyle/ |
| 7 | (fitness or exercis* or sport* or game* or aerobic* or run or runs or running or walk or walks or walking or jumping or outdoor* or bicycle* or biking or jog*).ti,ab. |
| 8 | (physical* adj2 (fit* or train* or endur* or activ* or inactivit* or energy)).ti,ab. |
| 9 | (exercis* adj2 (train* or physical* or activ*)).ti,ab. |
| 10 | (insufficient* adj2 (activ* or energy)).ti,ab. |
| 11 | ((lifestyle* or life-style*) **adj2** (activ* or physical* or healthy)).ti,ab. |
| 12 | 1 or 2 or 3 or 4 or 5 or 6 or 7 or 8 or 9 or 10 or 11 |
| 13 | exp "Diet, Food, and Nutrition"/ |
| 14 | exp nutrition disorders/ |
| 15 | (diet* or eat* or nutrition* or meal* or food* or lunch* or breakfast* or dinner* or supper or snack* or fruit* or vegetable* or sugar* or salt* or appetite* or overeat* or over-eat* or overnutrit* or over-nutrit* or overweight or over-weight or obese or obesity or overnourish* or over-nourish*).ti,ab. |
| 16 | 13 or 14 or 15 |
| 17 | exp sedentary lifestyle/ |
| 18 | Leisure activities/ |
| 19 | exp relaxation/ |
| 20 | exp hobbies/ |
| 21 | exp "play and playthings"/ |
| 22 | (sedentary adj2 (lifestyle* or life-style* or behavior* or behaviour* or web brows*)).ti,ab. |
| 23 | ((watch*) adj3 (television OR TV))).ti,ab. |
| 24 | ((video game* adj3 play*) or video gaming).ti,ab. |
| 25 | ((electronic* or computer* or media) adj2 (game* or us* or play* or time*)).ti,ab. |
| 26 | (screen adj2 (time* or recreation* or view* or activit*)).ti,ab. |
| 27 | 17 or 18 or 19 or 20 or 21 or 22 or 23 or 24 or 25 or 26 |
| 28 | exp "Tobacco Use Disorder"/ |
| 29 | exp smoking/ |
| 30 | (cigarette* or smok* or tobacco or vaping or vape* or eCig* or e-cig* or electronic cig*).ti,ab. |
| 31 | 28 or 29 or 30 |
| 32 | exp Drinking Behavior/ |
| 33 | (alcoholic* or intoxication or liquor or hangover*).ti,ab. |
| 34 | (alcohol* adj3 (abus* or us* or disorder* or consum* or drink* or heav* or problem* or excess* or bing* or risk* or reckless*)).ti,ab. |
| 35 | (drink* adj3 (alcohol* or abus* or us* or disorder* or consum* or heav* or problem* or excess* or bing* or risk* or reckless*)).ti,ab. |
| 36 | 32 or 33 or 34 or 35 |
| 37 | exp sleep/ |
| 38 | (sleep* or insomnia* or wakeful* or restless* or Chronotype).ti,ab. |
| 39 | 37 or 38 |
| 40 | 12 or 18 or 27 or 31 or 36 or 39 |
| 41 | exp Cell Phones/ |
| 42 | exp Smartphone/ |
| 43 | exp Mobile Applications/ |
| 44 | ((cell* or smart* or mobile or Google OR sidekick) adj3 (app or application or apps or applications or phone*)).ti,ab. |
| 45 | (smartphone* or sidekick or android* or iphone*).ti,ab. |
| 46 | ((smart phone* or cell* phone* or mobile phone* or application or applications or app or apps) adj3 (fitbit or fitbits or pedomet* or actigraphy or acceleromet* or mHealth or m-health or mobile health or e-health or "electronic health" or ehealth or telehealth or tele-health)).ti,ab. |
| 47 | 41 or 42 or 43 or 44 or 45 or 46 |
| 48 | exp "Outcome Assessment (Health Care)"/ |
| 49 | exp Observer Variation/ |
| 50 | exp Health Status Indicators/ |
| 51 | exp "Reproducibility of Results"/ |
| 52 | exp Discriminant Analysis/ |
| 53 | (instrumentation or methods).sh. |
| 54 | (Validation Studies or Comparative Study).pt. |
| 55 | "outcome assessment".ti,ab. |
| 56 | "outcome measure*".ti,ab. |
| 57 | "observer variation".ti,ab. |
| 58 | ((reproducib* or coefficient or homogeneity or homogeneous or "internal consistency" or error or errors or replicab* or repeated or variability or sensitiv*) adj5 (measure or measures or measurement or measurements or findings or finding or results or result or test or tests or testing or retest or instrument or instruments or instrumentation or scale or scaling or scales or analysis or analyses or value or values)).ti,ab. |
| 59 | (reliab* or unreliab* or valid*).ti,ab. |
| 60 | (cronbach* and (alpha or alphas)).ti,ab. |
| 61 | (item adj3 (correlation* or selection* or reduction*)).ti,ab. |
| 62 | (precision or imprecision or "precise values" or generaliza* or generalisa* or concordance or "item discriminant" or "interscale correlation*" or "individual variability").ti,ab. |
| 63 | (test* adj2 retest*).ti,ab. |
| 64 | (intraclass correlation*).ti,ab. |
| 65 | ((multitrait and scaling) and (analysis or analyses)).ti,ab. |
| 66 | ((uncertainty or uncertainties) adj2 (measurement or measuring)).ti,ab. |
| 67 | ((minimal or minimally or clinical or clinically) adj5 (important or importance or significant or significance or detectable or real) adj5 (change or difference)).ti,ab. |
| 68 | ("meaningful change" or "ceiling effect" or "floor effect" or "Item response model" or IRT or Rasch or "Differential item functioning" or DIF or "computer adaptive testing" or "item bank" or "cross-cultural equivalence").ti,ab. |
| 69 | 48 or 49 or 50 or 51 or 52 or 53 or 54 or 55 or 56 or 57 or 58 or 59 or 60 or 61 or 62 or 63 or 64 or 65 or 66 or 67 or 68 |
| 70 | 40 and 47 and 69 |
| 71 | limit 70 to yr="2007 - 2018" |
